# Qingfei Litan Decoction Against Acute Lung Injury/Acute Respiratory Distress Syndrome: The Potential Roles of Anti-Inflammatory and Anti-Oxidative Effects

**DOI:** 10.3389/fphar.2022.857502

**Published:** 2022-05-23

**Authors:** Yirui Diao, Qi Ding, Gonghao Xu, Yadong Li, Zhenqiu Li, Hanping Zhu, Wenxiang Zhu, Peng Wang, Yuanyuan Shi

**Affiliations:** ^1^ School of Life Sciences, Beijing University of Chinese Medicine, Beijing, China; ^2^ Shenzhen Research Institute, Beijing University of Chinese Medicine, Shenzhen, China; ^3^ Traditional Chinese Medicine, The First Affiliated Hospital of Guangzhou Medical University, Guangzhou, China; ^4^ School of Chinese Materia Medica, Beijing University of Chinese Medicine, Beijing, China

**Keywords:** systemic pharmacology, anti-inflammatory, anti-oxidative, acute lung injury, acute respiratory distress syndrome, traditional Chinese medicine

## Abstract

Acute lung injury/acute respiratory distress syndrome (ALI/ARDS) is an acute respiratory failure syndrome characterized by progressive arterial hypoxemia and dyspnea. Qingfei Litan (QFLT) decoction, as a classic prescription for the treatment of acute respiratory infections, is effective for the treatment of ALI/ARDS. In this study, the compounds, hub targets, and major pathways of QFLT in ALI/ARDS treatment were analyzed using Ultra high performance liquid chromatography coupled with mass spectrometry (UHPLC-MS) and systemic pharmacology strategies. UHPLC-MS identified 47 main components of QFLT. To explore its anti-inflammatory and anti-oxidative mechanisms, gene ontology (Go) analysis, Kyoto Encyclopedia of Genes and Genomes (KEGG) enrichment and network pharmacological analysis were conducted based on the main 47 components. KEGG enrichment analysis showed that TNF signaling pathway and Toll-like receptor signaling pathway may be the key pathways of ALI/ARDS. We explored the anti-inflammatory and anti-oxidative pharmacological effects of QFLT in treatment of ALI/ARDS *in vivo* and *in vitro*. QFLT suppressed the levels of proinflammatory cytokines and alleviated oxidative stress in LPS-challenged mice. *In vitro*, QFLT decreased the levels of TNF-α, IL-6, IL-1β secreted by LPS-activated macrophages, increased GSH level and decreased the LPS-activated reactive oxygen species (ROS) in lung epithelial A549 cells. This study suggested that QFLT may have anti-inflammatory and anti-oxidative effects on ALI/ARDS, combining *in vivo* and *in vitro* experiments with systemic pharmacology, providing a potential therapeutic strategy option.

## Introduction

Acute lung injury (ALI) and its serious form acute respiratory distress syndrome (ARDS) are considered acute respiratory failure syndrome, characterized by progressive arterial hypoxemia and dyspnea (Matthay and Zemans, 2011; Matthay et al., 2012; Sweeney and McAuley, 2016; Thompson et al., 2017). Several clinical diseases are known risk factors for ARDS, including pneumonia, sepsis, stomach contents aspiration, and major trauma (Matthay et al., 2012; Thompson et al., 2017). ARDS has a high mortality rate, with 28-day mortality reaching approximately 20%–40% (Ellani et al., 2016; Sweeney and McAuley, 2016). The Berlin definition for ARDS published in 2012 states that: based on hypoxemia, ARDS is classified as three levels, including mild (200 mm Hg < PaO_2_/FIO_2_ ≤ 300 mm Hg), moderate (100 mm Hg < PaO_2_/FIO_2_ ≤ 200 mm Hg), and severe (PaO_2_/FIO_2_ ≤ 100 mm Hg) (ARDS Definition Task Force et al., 2012; Sweeney and McAuley, 2016; Thompson et al., 2017). Despite decades of clinical trials, treatment options for ARDS are limited, and therapy is mainly supportive care with mechanical ventilation (Fan et al., 2005; Fan et al., 2018). Unfortunately, such as glucocorticoids, surfactants, inhaled nitric oxide, antioxidants, protease inhibitors, anticoagulation, non-steroidal anti-inflammatory agents, β2 agonists, statins, and albuterol (Matthay and Zemans, 2011; Thompson et al., 2017) remain controversial, which target pathophysiologic alterations in ARDS (Fan et al., 2018). Coronavirus 19 (COVID-19) has spread globally, and many patients with severe COVID-19 develop ARDS shortly after onset of dyspnea and hypoxemia (Berlin et al., 2020). Therefore, the development of new drugs for ALI/ARDS may also be beneficial for treatment of COVID-19. Furthermore, tissue damage mediated by inflammatory mediators and oxidants is an important event in the pathogenesis of ARDS (Tasaka et al., 2008; Lv et al., 2017). Pulmonary oxidative damage and inflammation enhances neutrophil permeability across the endothelial/epithelial barrier and releases cytotoxic factors such as proinflammatory cytokines and reactive oxygen species (ROS) (Kellner et al., 2017). Moreover, injury of essential biomolecules and cells results in excessive inflammatory response (Lei et al., 2015), which would exacerbate the tissue injury and pulmonary edema. Consequently, the inhibition of excess inflammation and oxidative stress would be a beneficial basic strategy for the prevention and treatment of ALI/ARDS.

It is widely known that traditional Chinese medicine (TCM) has various pharmacological properties (Li and Kan, 2017). Compared with western medicine, it often exerts a protective effect on ALI/ARDS through multiple targets. In addition, dexamethasone and prednisone, which are clinically used to treat ALI, may have anti-inflammatory effects on ARDS, but have multiple side effects, including immunosuppression, upper gastrointestinal bleeding, osteoporosis and myopathy (Mokra et al., 2019). It has been reported that many natural compounds may suppress ALI development due to their anti-inflammatory and anti-oxidative activities and relatively minor side effects. Furthermore, many traditional Chinese patent medicines such as Tanreqing injection, Lianhua Qingwen capsule, which contain various natural compounds, have a remarkable curative effect on COVID-19 *via* anti-inflammatory, anti-viral, and anti-lung injury properties (Zhuang et al., 2020). Moreover, the effect of integrated traditional Chinese and Western medicine has also been recognized (Ni et al., 2020). Therefore, TCM treatment may be a promising new option for ALI/ARDS.

Qingfei Litan (QFLT) decoction has been included in the *Wind-Heat in Lung Syndrome TCM Diagnosis and Treatment Plan* by the TCM State Administration. As a classic prescription for the treatment of acute respiratory infections, it was applied in the middle stage of Wind-Heat in Lung Syndrome. It is believed to have the effects of removing heat-phlegm, dispersing lung, and relieving cough in TCM clinical prescriptions. QFLT has been effective in ALI/ARDS clinical treatment for years, and it has been reported that combined QFLT and Western medicine is more effective in the treatment of pneumonia cough than Western medicine alone (Zeng et al., 2012). QFLT has also been found to have significant effects on significantly lower symptom scores and improved pulmonary function indices in patients with COPD exacerbations (Zhu et al., 2019). However, the exact active compounds, targets, and the underlying mechanisms remain unclear.

As an emerging discipline combining experimental analysis and computational tools, systems pharmacology can not only increase the understanding of the treatment mechanisms of complex diseases (Hopkins, 2008; Fang et al., 2018), but also reveal optimal TCM prescriptions as well as better reflecting the integrity of TCM (Ding et al., 2020; Zhao et al., 2020).

**FIGURE 1:**
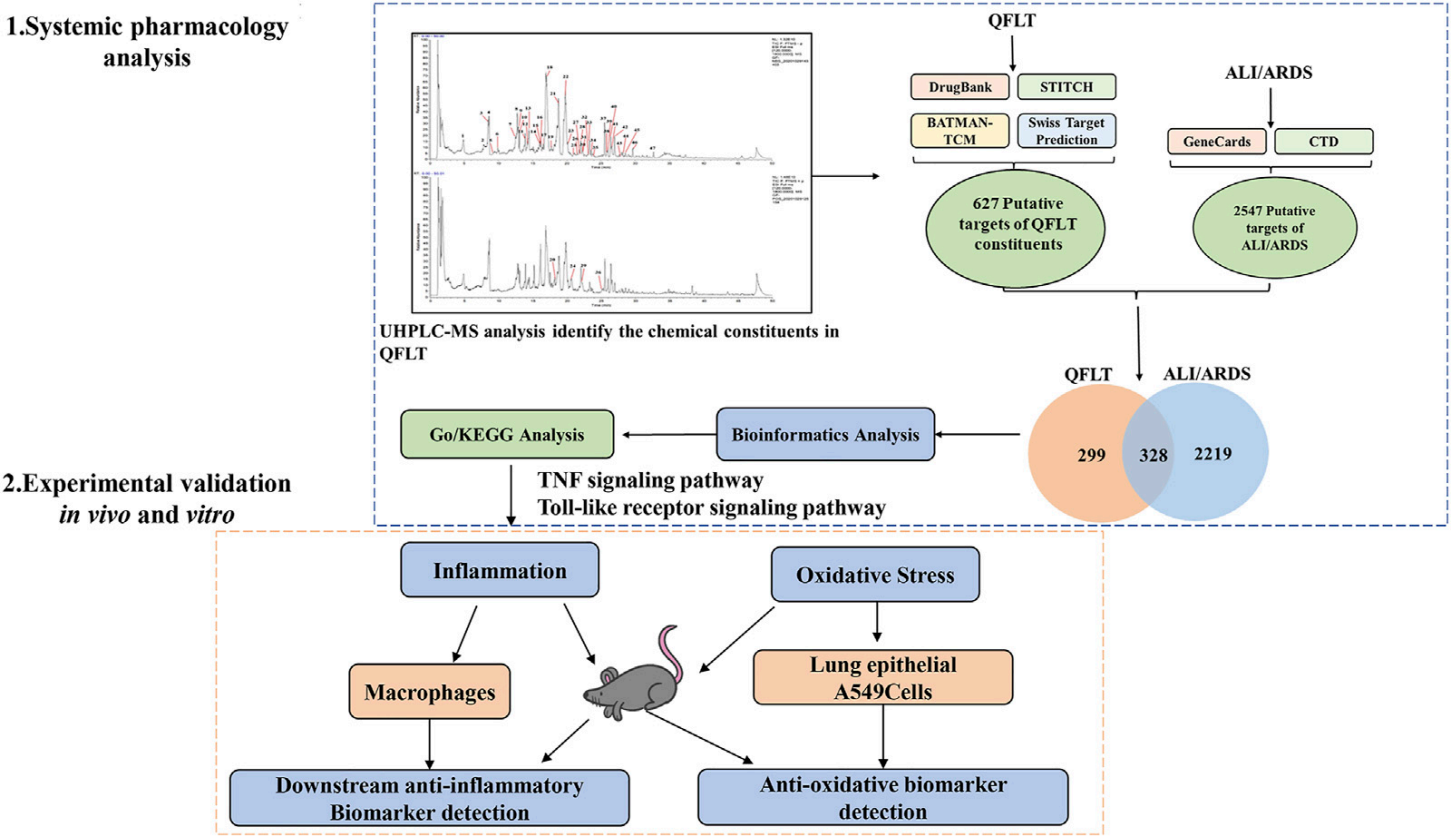
Procedures of a systemic pharmacology-based strategy to investigate the effect of Qingfei Litan (QFLT) decoction against Acute lung injury (ALI)/acute respiratory distress syndrome (ARDS).

In this study (Figure 1), based on UHPLC-MS and systemic pharmacology strategies, we analyzed the compounds, hub targets, and essential pathways of QFLT in ALI/ARDS treatment. Combined with previous studies and KEGG enrichment, TNF signaling pathway, Toll-like receptor signaling pathway and Nrf2 signaling pathway were likely to be the potential anti-inflammatory and anti-oxidative mechanisms of QFLT. Therefore, downstream biomarkers (TNF-α, IL-1β, IL-6, SOD, MDA, GSH, ROS, GSH-Px) of these pathways were verified *in vivo* and *in vitro* experiments. Our experiments confirmed that QFLT has significant pharmacological effects in regulating inflammatory mediators and oxidative stress levels in LPS-challenged mice. *In vitro*, QFLT decreased the levels of tumor necrosis factor alpha (TNF-α), interleukin-6 (IL-6), interleukin-1β (IL-1β) secreted by macrophages and reduced oxidation production of lung epithelial A549 cells. From the perspective of network pharmacology and experimental verification, the results revealed the potential anti-inflammatory and anti-oxidative mechanisms of QFLT in the treatment of ALI/ARDS.

## Material and Methods

### Preparation of QFLT Decoction

QFLT decoction consisted of CaSO_4_·2H_2_O (Gypsum fibrosum) (Shigao, SG) 30 g, *Nervilia fordii* (Hance) Schltr. (Orchidaceae, Nervilia plicatae herba) (Qingtiankui, QTK) 10 g, *Trichosanthes kirilowii* Maxim. (Cucurbitaceae, Trichosanthis pericarpium) (Gualoupi, GLP) 15 g, *Scutellaria baicalensis* Georgi (Lamiaceae, Scutellariae radix) (Huangqin, HQ) 15 g, *Fritillaria thunbergii* Miq. (Liliaceae, Fritillariae thunbergii bulbus) (Zhebeimu, ZBM) 15 g, *Houttuynia cordata* Thunb. (Saururaceae, Houttuyniae herba) (Yuxingcao, YXC) 20 g, *Prunus armeniaca* L. (Rosaceae, Armeniacae semen amarum) (Kuxingren, KXR) 10 g, *Platycodon grandiflorus* (Jacq.) A. DC. (Campanulaceae, Platycodonis radix) (Jiegeng, JG) 15 g, *Phragmites australis* (Cav.) Trin. ex [Poaceae, Phragmitis rhizoma] (Lugen, LG) 15 g and *Glycyrrhiza uralensis* Fisch. ex DC. (Fabaceae, Glycyrrhizae radix et rhizoma) (Gancao, GC) 10 g. These components were purchased from the First Affiliated Hospital of Guangzhou Medical University. Before the experiment, the Chinese medicine extract was made by Wuhan Kangle Pharmaceutical Co., Ltd. A total of 155 g (raw materials) QFLT decoction was soaked in 1,500 ml distilled water for 1 h, then decocted for 2 h (CaSO_4_·2H_2_O first decocted for 30 min) and filtered. The filter residues were soaked in 750 ml of distilled water, boiled for 2 h, and filtered again (Liu et al., 2014). Then, we obtained about 200 ml of filtrates each time. And the two filtrates were combined to obtain 400 ml of QFLT solution. The preparation method was consistent with the process used for patients in medicine/hospital. The combined filtrates were concentrated to 100 ml, then freeze-dried to obtain the extract (extraction rate 26%), and stored at −20°C. The dried powder was dissolved in water to prepare oral QFLT for mice with three concentrations of 0.4, 0.8, and 1.6 g/ml, and stored at −4°C.

### UHPLC and MS Analysis

For analysis, the chemical composition of QFLT was determined using the Thermo Dionex Ultimate 3000 UPLC system (Thermo Fisher, Waltham, MA, United States). 1 g QFLT freeze-dried powder was added to 10 ml chromatographic methanol, and ultrasonically extracted for 1 h. The extract was centrifuged at 12,000 rpm for 15 min and then passed through a 0.22 μm microporous membrane. The QFLT sample was separated on a Waters Acquity UPLC BDS C18 column (2.4 × 150 mm, 2.74 μm). The sample injection volume was 5 μL. In the mobile phase, the mixture of acetonitrile (A) and 0.1% formic acid (B) as solvent performed to elute at a flow rate of 0.30 ml/min. Column temperature was set at 45°C. The gradient elution was 0–3 min, 95% B; 3–45 min, 95%–25% B; 45.1–50 min, 95% B. Mass conditions were set as follows: spray voltage (+), 3.5 kV; spray voltage (−), 3.0 kV; capillary temperature, 320°C; sheath gas flow, 30 Arb; aux gas flow, 10 Arb; aux gas heater temperature, 400°C; and S-Lens RF Level, 55%. Finally, the total ion chromatogram was obtained under the positive and negative ion mode. Subsequently, according to published indicators of quality, chromatographic behavior and fragment ion mass, the chemical compounds in QFLT were identified. The data were processed using Xcalibur software version 2.7.

### Putative Targets Prediction of Components Identified in QFLT

We collected the putative targets of QFLT using SwissTargetPrediction (http://www.swisstargetprediction.ch/), BATMAN-TCM (http://bionet.ncpsb.org.cn/batman-tcm/), STITCH v5.0 195 (http://stitch.embl.de/) and Drugbank v5.1.7 196 (https://go.drugbank.com/). Gene names corresponding to protein names were identified by Uniprot (https://www.uniprot.org/).

### Visualizing the Protein–Protein Interaction Network

In String v11.0, we searched these putative targets using the multiple proteins option. We set the organism to be homo sapiens and selected a high confidence level (0.700). We performed PPI network visualization and further analysis using Cytoscape v3.7.2. In addition, the cytohubba app of Cytoscape was utilized to analyze the PPI network.

### Acquisition of ALI/ARDS-Related Targets

We collected disease gene targets for ALI/ARDS from two databases, GeneCards (https://www.genecards.org/) and CTD (https://ctdbase.org/). The search terms “acute lung injury” or “acute respiratory distress syndrome” were employed to acquire ALI/ARDS-related targets. After deletion of redundant items, the Venn diagram showing interaction between QFLT and ALI/ARDS was created.

### Enrichment of GO/KEGG Pathways

GO/KEGG Pathways enrichment analysis relied on the Gene Ontology (GO) and Kyoto Encyclopedia of Genes and Genomes (KEGG) database by DAVID v6.8, in which *p* < 0.05 was considered statistically significant.

### Animals

Seven-week-old adult male mice C57BL/6J of average weight 18–22 g were purchased from Weitong Lihua Laboratory Animal Technology Co., Ltd. [Animal License: SCXK (Jing) 2016-0006] in Beijing, China. They were raised in an environment matching SPF-condition for several days. The number of animals in each group was estimated based on previous studies of LPS-challenged ALI animal models (Fu et al., 2019; Amatullah et al., 2021). The methods of animal experiments followed the ethical codes of Beijing University of Chinese Medicine. The experiments have been approved by the Animal Care and Use Committee of Beijing University of Chinese Medicine (BUCM-4-2019102105-4119).

### Animals Group and Treatment

Mice were randomly divided into six groups with eight mice in each group, including the control, LPS, low-dose, medium-dose, high-dose, and control-high-dose groups. After a 12-h fasting, mice were treated with LPS (5 mg/kg) (Sigma-Aldrich, St. Louis, Missouri) in the LPS group and 0.9% NaCl in the control group (Zhou et al., 2017; Zhong et al., 2019; Huang et al., 2020) One week before intratracheal administration, mice were treated with low-dose QFLT (4 g/kg), medium-dose QFLT (8 g/kg), high-dose QFLT (16 g/kg), control-high-dose QFLT (16 g/kg) once a day as a protective treatment. The control and LPS groups were orally administered equal volumes of 0.9% NaCl.

Dosage of QFLT decoction was determined according to conversions from clinical daily adult dosages. The daily dosage of QFLT for mice is 8 g/kg (the extract) for mice, equivalently, the daily dose of QFLT for adult is 40.3 g (the extract), which is calculated based on formula converting dosage of human into the mouse according to the respective body surface areas of the Chinese Medicine Pharmacology Research Technology. In order to explore the dose-effect relationship, doubled up as the high-dose group (16 g/kg), and doubled down as the low-dose group (4 g/kg). Therefore, this study set the dose of QFLT (4, 8, and 16 g/kg) to treat ALI mice.

Twenty-four hours after LPS administration, mice were euthanized using sodium pentobarbital injection. Bronchoalveolar lavage fluid (BALF) and lung tissues were collected for further studies. BALF was stained using Wright-Giemsa solution (Solarbio, Beijing, China) to sort and count the cells. The upper left lung was excised and fixed with 4% neutral formaldehyde for conventional hematoxylin and eosin (H&E) staining to reflect the pathological morphological changes in the lung tissue of LPS-challenged mice. ALI H&E sections of 4–5 μm in thickness were examined and assessed by professionals under light microscopy. Lesion severity was scored as: 0 (normal), 0.5 (mild), 1 (mild), 2 (moderate), 3 (severe), based on indicators including alveolar wall congestion, emphysema, bronchial epithelial cell degeneration, inflammatory cell infiltration and lymphocyte proliferation. The right lower lung’s wet/dry (W/D) weight ratio was calculated to evaluate lung edema.

### Enzyme-Linked Immunosorbent Assay

The Enzyme-linked immunosorbent assay kit (ELISA) theory utilized specific antibody-antigen interactions to detect antigen. We detected the secretion of inflammatory cytokines TNF-α, IL-6 and IL-1β in BALF using an ELISA kit (Proteintech, Wuhan, China). In addition, we collected the macrophages cell-free supernatants for the detection of TNF-α, IL-6, and IL-1β secretion. The optical density was measured at 450 nm.

### Reverse-Transcription Quantitative Polymerase Chain Reaction

To quantify cytokines RNA expression in mice lungs stimulated with LPS, the levels of *TNF-α, IL-1β, IL-6* mRNA were examined using qPCR. We used the NCBI database for primers design and purchased primers from Sangon Biotech (Shanghai, China). The primers were presented as follows. Data were normalized by the expression of GAPDH mRNA in each sample. *TNF-α* (Forward primer: GAT​C-GGT​CCC​CAA​AGG​GAT​G, Reverse primer: CCA​CTT​GGT​GGT​TTG​TGA​GTG), *IL-1β* (Forward primer: AGA​GTC​CCC​AAC​TCA​TCT​CCT, Reverse primer: AAG​TCC​CTA​GGT​TGG​GCT​TG), *IL-6* (Forward primer: GAC​AAA​GCC​AGA​GTC​CTT​CAG​A, Reverse primer: TGT​GAC​TCC​AGC​TTA​TCT​CTT​GG), *GAPDH* (Forward primer: CTC​TGG​TGG​CTA​GCT​CAG​AAA, Reverse primer: CCC​TGT​TGC​TGT​AGC​CGT​AT).

### Oxidation Product Assay

The multiple oxidation products in mice lungs and A549 cells were measured using the MDA assay kit (TBA method), SOD assay kit (hydroxylamine method), GSH-Px assay kit (colorimetric method) and GSH kit, respectively (Nanjing, China). The ROS was evaluated using a Fluorometric Intracellular ROS kit (Beyotime, China).

### Cell Culture and Treatment

Macrophages (MH-S) (Manassas, VA, United States) were incubated in DMEM containing 10% FBS fetal bovine serum, 100 units/mL Penicillin G and 100 μg/ml Streptomycin Sulfate solution at 37°C in a 5% CO_2_ atmosphere. The cells were then cultured in a serum-free medium for 3 h and stimulated with LPS (5 μg/ml) in the presence or absence of QFLT. After 24-h incubation, the cultured medium was used for cytokine detection with the ELISA kit.

The human lung type II epithelial cell line A549 (Stem Cell Bank, Chinese Academy of Sciences, Shanghai, China) was incubated in a DMEM medium containing 10% FBS and 1%PS. Cells were grown in a 96-well plate to 70%–80% confluency overnight and then in a serum-free medium for 3 h. After QFLT (0.1, 1, 10 μg/ml) pretreatment for 24 h, cells were incubated with LPS (800 μg/ml) for 24 h to detect cell viability by CCK-8.

### Statistical Analyses

All data referenced above experiments were calculated as the mean ± SD. Each assay was performed for at least three independent and repeatable experiments. The statistical analysis and graphics were performed using GraphPad Prism 8.0. One-way ANOVA and Student’s two-tailed t-test were carried out. *p* < 0.05 was considered to indicate a statistically significant difference between groups.

## Results

### UHPLC-MS Analysis of the Chemical Compounds of QFLT

**FIGURE 2:**
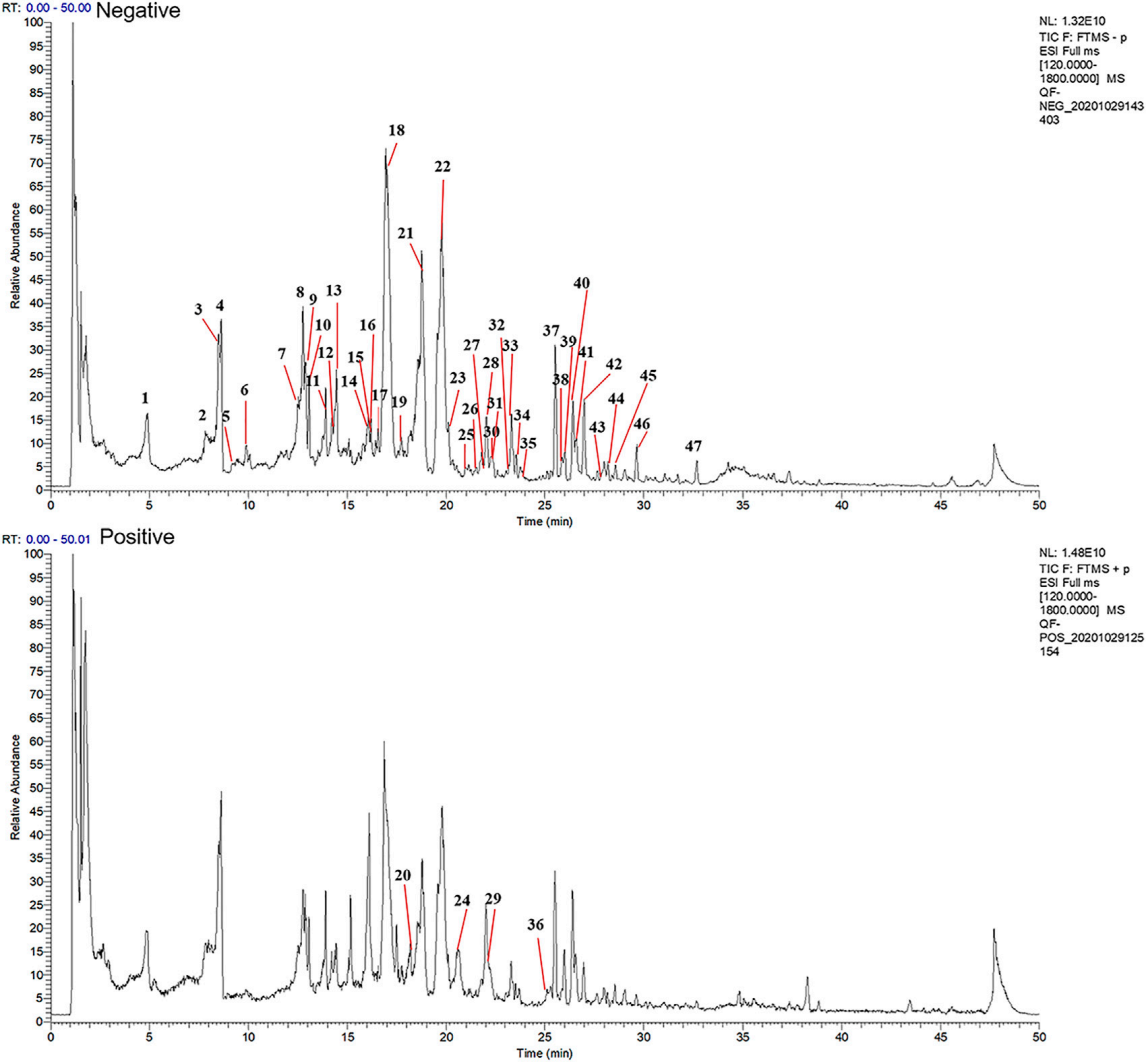
Total ion chromatogram monitored in negative and positive ion modes for QFLT extract.

Aqueous extract of QFLT was analyzed by UHPLC-MS to identify the chemical compounds (Figure 2). Based on the retention time and molecular ion peaks, compared with published data from TCMSP database (http://tcmspw.com/tcmsp.php), accurate quality, chromatographic behavior, and fragment ion mass, 47 chemical components were identified, including 35 flavonoids, four alkaloids, four organic acids, three cyanogenic glycosides, and one coumarin, (Table 1).

**TABLE 1:** Identified compounds of QFLT by UHPLC-MS.

No	RT	Compound	ESI	MS	Molecular formular	Molecular weight	Error/ppm	CID	Source	MS/MS
1	4.90	Hopantenic acid	[M-H]^−^	232.1191	C_10_H_19_NO_5_	233.1263	2.573	28281	LG	102.0562 [M-H-C_6_H_11_O_4_N]^−^, 146.0827 [M-H-C_4_H_6_O_2_]^−^
2	7.91	Cryptochlorogenic acid	[M-H]^−^	353.0883	C_16_H_18_O_9_	354.0951	2.824	9798666	YXC	173.0458 [M-H-C_9_H_8_O_4_]^−^, 179.0353 [M-H-C_7_H_10_O_5_]^−^, 191.0565 [M-H-C_9_H_6_O_3_]^−^
3	8.50	Neoamygdalin	[M + HCOO]^−^	502.1575	C_20_H_27_NO_11_	457.1584	3.062	441462	KXR	161.0459 [M + HCOO-C_15_H_19_O_8_N]^−^, 221.0673 [M + HCOO-C_13_H_15_O_5_N]^−^,323.0987 [M + HCOO-C_9_H_9_O_3_N]^−^
4	8.63	Amygdalin	[M + HCOO]^−^	502.1573	C_20_H_27_NO_11_	457.1584	2.624	656516	KXR	221.0673 [M + HCOO-C_13_H_15_O_6_N]^−^, 263.0779 [M + HCOO-C_11_H_13_O_5_N]^−^
5	9.20	Taxifolin	[M-H]^−^	303.0516	C_15_H_12_O_7_	304.0583	3.617	439533	JG	125.0247 [M-H-C_9_H_6_O_4_]^−^, 177.0197 [M-H-C_6_H_6_O_3_]^−^, 217.0512 [M-H-C_3_H_2_O_3_]^−^
6	9.90	Prunasin	[M-H]^−^	294.0991	C_14_H_17_NO_6_	295.1056	4.405	119033	KXR	71.014 [M-H-C_12_H_18_O_3_]^−^, 85.0297 [M-H-C_11_H_16_O_3_]^−^,161.0459 [M-H-C_9_H_12_]^−^
7	12.54	Isoliquiritin	[M-H]^−^	417.1197	C_21_H_22_O_9_	418.1264	2.630	5318591	GC	135.0091 [M-H-C_14_H_18_O_6_]^−^, 255.0670 [M-H-C_6_H_10_O_5_]^−^
8	12.77	Liquiritin	[M-H]^−^	417.1195	C_21_H_22_O_9_	418.1264	2.152	503737	GC	135.0090 [M-H-C_14_H_18_O_6_]^−^, 255.0670 [M-H-C_6_H_10_O_5_]^−^
9	12.88	Licraside	[M-H]^−^	549.1620	C_26_H_30_O_13_	550.1686	2.181	14282455	GC	135.0090 [M-H-C_19_H_26_O_10_]^−^, 255.0668 [M-H-C_11_H_18_O_9_]^−^
10	13.07	Chrysin-6-C-Arabinoside 8-C-Glucoside	[M-H]^−^	547.1464	C_26_H_28_O_13_	548.153	2.189	73829976	HQ	337.0728 [M-H-C_7_H_14_O_7_]^−^,367.0832 [M-H-C_6_H_12_O_6_]^−^, 457.1154 [M-H-C_3_H_6_O_3_]^−^
11	13.92	Chrysin 6-C-glucoside-8-C-alpha-L-arabinopyranoside	[M-H]^−^	547.1464	C_26_H_28_O_13_	548.153	2.189	44257617	HQ	337.0728 [M-H-C_7_H_14_O_7_]^−^, 367.0833 [M-H-C_6_H_12_O_6_]^−^, 427.1046 [M-H-C_3_H_8_O_4_]^−^, 457.1154 [M-HC_3_H_6_O_3_]^−^, 547.1474 [M-H-H_2_O]^−^
12	14.27	Luteolin	[M-H]^−^	285.0407	C_15_H_10_O_6_	286.0477	2.796	5280445	JG	151.0039 [M-H-C_8_H_6_O_2_]^−^,199.0403 [M-H-C_3_H_2_O_2_]^−^, 241.0511 [M-H-CO_2_]^−^
13	14.43	Quercitrin	[M-H]^−^	447.0937	C_21_H_20_O_11_	448.1006	2.008	5280459	YXC	300.0282 [M-H-C_6_H_11_O_4_]^−^,301.0360 [M-H-C_6_H_10_O_4_]^−^
14	16.05	Kaempferide 3-Glucoside	[M-H]^−^	461.1096	C_22_H_22_O_11_	462.1162	2.596	44259083	YXC	298.0491 [M-H-C_6_H_11_O_5_]^−^, 271.0620 [M-H-C_7_H_10_O_6_]^−^
15	16.10	Complanatuside	[M + HCOO]^−^	669.1687	C_28_H_32_O_16_	624.169	3.204	5492406	QTK	298.0490 [M + HCOO-C_13_H_23_O_12_]^−^, 299.0569 [M + HCOO-C_13_H_22_O_12_]^−^
16	16.19	Licuroside	[M-H]^−^	549.1622	C_26_H_30_O_13_	550.1686	2.544	6475724	GC	135.0091 [M-H-C_19_H_26_O_10_]^−^, 255.0671 [M-H-C_11_H_18_O_9_]^−^
17	16.56	Neoisoliquiritin	[M-H]^−^	417.1200	C_21_H_22_O_9_	418.1264	3.348	5320092	GC	255.0668 [M-H-C_6_H_10_O_5_]^−^, 135.0090 [M-H-C_14_H_18_O_6_]^−^
18	17.00	Baicalin	[M-H]^−^	445.0779	C_21_H_18_O_11_	446.0849	1.793	64982	HQ	85.0297 [M-H-C_17_H_12_O_9_]^−^, 113.0247 [M-H-C_16_H_12_O_8_]^−^, 269.0461 [M-H-C_6_H_8_O_6_]^−^
19	17.71	Dihydrobaicalein 7-O-glucuronide	[M-H]^−^	447.0940	C_21_H_20_O_11_	448.1006	2.677	14135324	HQ	113.0247 [M-H-C_5_H_5_O_3_]^−^, 243.0669 [M-H-C_7_H_8_O_7_]^−^, 271.0620 [M-H-C_6_H_8_O_6_]^−^
20	18.19	Edpetiline	[M + H]^+^	592.3849	C_33_H_53_NO_8_	591.3771	0	90479257	ZBM	574.3745 [M + H-H_2_O]^+^
21	18.77	Wogonoside	[M-H]−	459.0938	C_22_H_20_O_11_	460.1006	2.173	3084961	HQ	268.0385 [M-H-C_7_H_11_O_6_]^−^, 283.0620 [M-H-C_6_H_8_-O_6_]^−^
22	19.79	Oroxylin A Glucoronide	[M-H]−	459.0938	C_22_H_20_O_11_	460.1006	2.173	14655552	HQ	268.0381 [M-H-C_7_H_11_O_6_]^−^, 283.0617 [M-H-C_6_H_8_O_6_]^−^
23	20.13	Diosmetin	[M-H]−	299.0563	C_16_H_12_O_6_	300.0634	2.332840326	5281612	GLP	211.0408 [M-H-C_3_H_4_O_3_]^−^, 283.0252 [M-H-CH_4_]^−^, 284.0336 [M-H-CH_3_]^−^
24	20.59	Peimine	[M + H]^+^	432.3475	C_27_H_45_NO_3_	431.3399	−0.463	131900	ZBM	414.3370 [M + H-H_2_O]^+^
25	20.96	Norwogonin	[M-H]−	269.0460	C_15_H_10_O_5_	270.0528	3.702	5281674	HQ	197.0611 [M-H-C_2_O_3_]^−^, 241.0513 [M-H-CO]^−^, 225.0565 [M-H-CO_2_]^−^
26	21.51	Rhamnazin	[M-H]−	329.0672	C_17_H_14_O_7_	330.074	3.029	5320945	QTK	165.9910 [M-H-C_7_H_2_O_5_]^−^, 314.0440 [M-H-CH_3_]^−^
27	21.87	Tricin	[M-H]−	329.0671	C_17_H_14_O_7_	330.074	2.726	5281702	LG	299.0203 [M-HC_15_H_7_O_7_]^−^, 314.0443 [M-H-CH_3_]^−^
28	22.04	Baicalein	[M-H]−	269.0458	C_15_H_10_O_5_	270.0528	2.962	5281605	HQ	241.0517 [M-H-CO]^−^, 251.0357 [M-H-H_2_O]^−^
29	22.12	Peiminine	[M + H]^+^	430.3318	C_27_H_43_NO_3_	429.3243	-0.698	167691	ZBM	412.3213 [M + H-H_2_O]^+^
30	22.31	Liquiritigenin	[M-H]^−^	255.0667	C_15_H_12_O_4_	256.0736	3.514	114829	GC	91.0191 [M-H-C_9_H_8_O_3_]^−^, 119.0504 [M-H-C_7_H_4_O_3_]^−^
31	22.36	Isoliquiritigenin	[M-H]^−^	255.0666	C_15_H_12_O_4_	256.0736	3.124	638278	GC	135.0090 [M-H-C_8_H_8_O_3_]^−^, 153.0197 [M-H-C_8_H_6_]^−^
32	23.06	Formononetin	[M-H]^−^	267.0667	C_16_H_12_O_4_	268.0736	3.357	5280378	GC	239.0357 [M-H-C_2_H_4_]^−^, 252.0434 [M-H-CH_3_]^−^
33	23.30	Pinellic Acid	[M-H]^−^	329.2336	C_18_H_34_O_5_	330.2406	2.422	9858729	QTK	211.1344 [M-H-C_6_H_14_O_2_]^−^, 229.1449 [M-H-C_12_H_21_O_4_]^−^
34	23.56	Tianshic Acid	[M-H]^−^	329.2338	C_18_H_34_O_5_	330.2406	3.028	5321949	QTK	171.1030 [M-H-C_9_H_18_O_2_]^−^, 211.1344 [M-H-C_6_H_14_O_2_]^−^, 229.1449 [M-H-C_6_H_12_O]^−^, 311.2233 [M-H-H_2_O]^−^
35	23.86	Isorhamnetin	[M-H]^−^	315.0516	C_16_H_12_O_7_	316.0583	3.480	5281654	GC	121.0296 [M-H-C_9_H_6_O_5_]^−^, 165.0196 [M-H-C_8_H_6_O_3_]^−^, 300.0281 [M-H-CH_3_]^−^
36	25.18	Isopeimine	[M + H]^+^	432.3475	C_27_H_45_NO_3_	431.3399	−0.463	21573744	ZBM	414.3372 [M + H-H_2_O]^+^
37	25.52	Wogonin	[M-H]^−^	283.0612	C_16_H_12_O_5_	284.0685	1.760	5281703	HQ	137.0249 [M-H-C_9_H_6_O_2_]^−^, 163.0035 [M-H-C_8_H_8_O]^−^, 268.0383 [M-H-CH_3_]^−^
38	25.86	Chrysin	[M-H]^−^	253.0509	C_15_H_10_O_4_	254.0579	3.148	5281607	HQ	107.0144 [M-H-C_9_H_6_O_2_]^−^, 209.0609 [M-H-CO_2_]^−^
39	26.01	Cirsimaritin	[M-H]^−^	313.0721	C_17_H_14_O_6_	314.079	2.865	188323	GLP	283.0254 [M-H-C_2_H_6_]^−^, 298.0490 [M-H-CH_3_]^−^
40	26.41	Casticin	[M-H]^−^	373.0931	C_19_H_18_O_8_	374.1002	1.871	5315263	QTK	343.0467 [M-H-C_2_H_6_]^−^, 358.0703 [M-H-CH_3_]^−^
41	26.57	Acacetin	[M-H]^−^	283.0614	C_16_H_12_O_5_	284.0685	2.464	5280442	JG	268.0385 [M-H-CH_3_]^−^
42	26.99	Kaempferide	[M-H]^−^	299.0562	C_16_H_12_O_6_	300.0634	1.999	5281666	YXC	93.0347 [M-H-C_6_H_5_O]^−^, 165.0197 [M-H-C_8_H_6_O_2_]^−^, 240.0435 [M-H-C_2_H_3_O_2_]^−^, 271.0619 [M-H-CO]^−^, 284.0330 [M-H-CH_3_]^−^
43	27.86	Sigmoidin B	[M-H]^−^	355.1194	C_20_H_20_O_6_	356.126	3.369	73205	GC	125.0246 [M-H-C_14_H_14_O_3_]^−^, 203.1082 [M-H-C_7_H_4_O_4_]^−^, 229.0875 [M-H-C_6_H_6_O_3_]^−^
44	28.16	Glycycoumarin	[M-H]^−^	367.1190	C_21_H_20_O_6_	368.126	2.173	5317756	GC	297.0412 [M-H-C_5_H_10_]^−^, 309.0414 [M-H-C_4_H_10_]^−^, 352.0961 [M-H-CH_3_]^−^
45	28.56	Glyasperin C	[M-H]^−^	355.1556	C_21_H_24_O_5_	356.1624	2.807	480859	GC	109.0297 [M-H-C_15_H_18_O_3_]^−^, 135.0454 [M-H-C_13_H_16_O_3_]^−^,151.0039 [M-H-C_14_H_20_O]^−^, 207.1033 [M-H-C_9_H_8_O_2_]^−^, 233.1187 [M-H-C_7_H_6_O_2_]^−^, 254.0590 [M-H-C_6_H_13_O]^−^, 323.1301 [M-H-CH_4_O]^−^
46	29.64	Glyasperin F	[M-H]^−^	353.1033	C_20_H_18_O_6_	354.1103	2.259	392442	GC	125.0246 [M-H-C_14_H_12_O_3_]^−^, 285.1139 [M-H-C_3_O_2_]^−^
47	32.69	Licoisoflavone B	[M-H]^−^	351.0877	C_20_H_16_O_6_	352.0947	2.272	5481234	GC	283.0981 [M-H-C_3_O_2_]^−^, 336.0654 [M-H-CH_3_]^−^

### Acquisition of Putative Targets of Compounds and Disease

**FIGURE 3:**
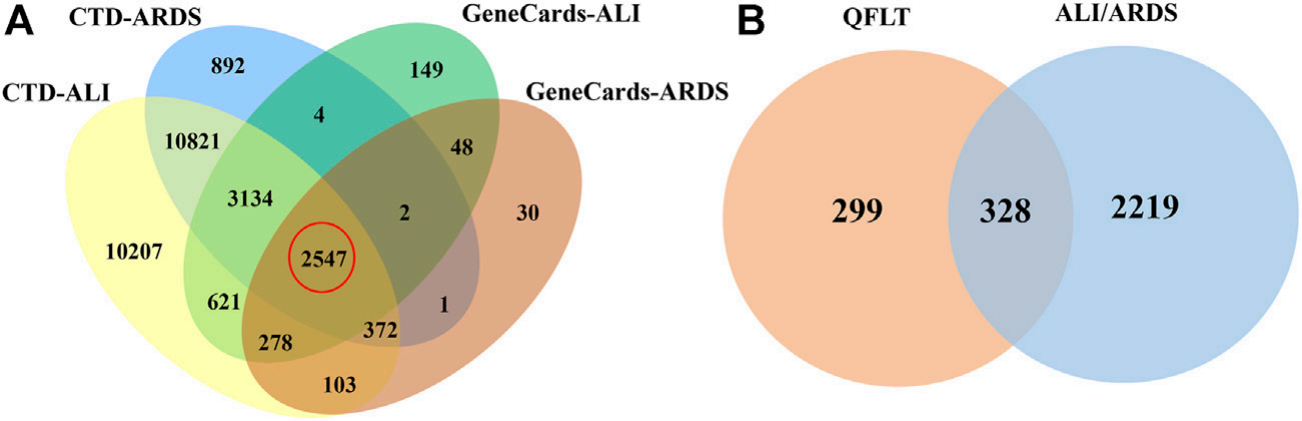
Venn diagram. **(A)** ALI/ARDS putative targets collected from two databases. **(B)** potential overlapping targets between QFLT and ALI/ARDS.

Compound targets collection relied on SwissTargetPrediction, BATMAN-TCM, STITCH and Drugbank database. After excluding duplicate values, 627 potentially relevant targets of QFLT were obtained. The detailed information about the interaction between potential targets and the 47 identified compounds was showed in Supplementary Data Sheet S1. Disease targets (Supplementary Data Sheet S2) collection identified 28,080 ALI-related targets and 17,773 ARDS-related targets in the CTD database and 6783 ALI-related targets and 3381 ARDS-related targets in the GeneCards database. Of these, 2547 overlapping targets may be considered important ALI/ARDS-related targets (Figure 3A). Figure 3B showed that 2547 targets for disease and 627 targets for QFLT had 328 overlaps (Supplementary Data Sheet S3), which may be the key targets for QFLT in treating ALI/ARDS.

### Revealing the QFLT’s Treatment on ALI/ARDS From the Network Level

**FIGURE 4:**
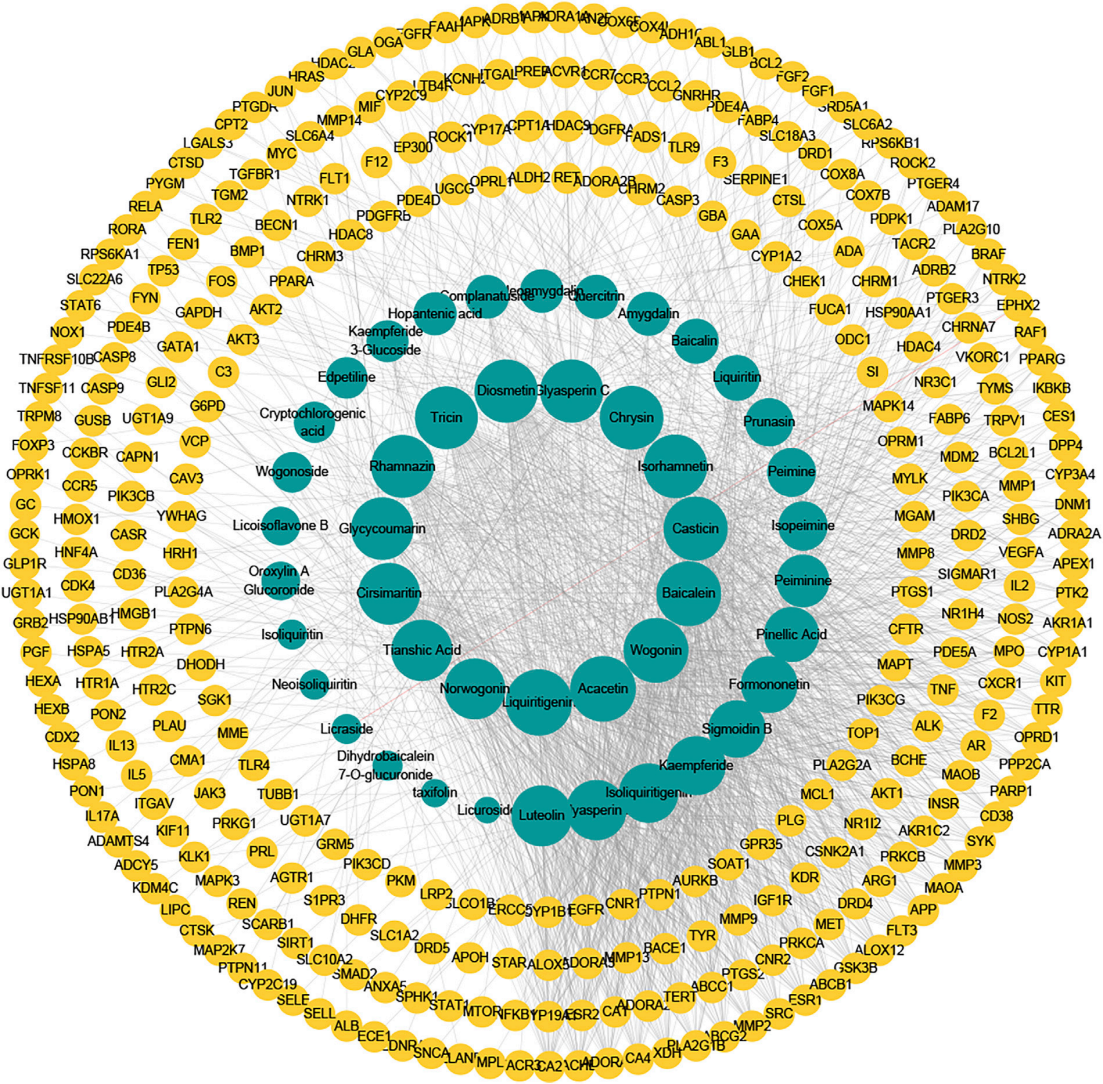
Compound–target network. The potentially important targets and compounds may be found, which may play roles in immune and inflammatory response, and oxidative stress.

Based on the 328 overlapping targets, we constructed the compound-target network (Figure 4). The network consisted of 373 nodes and 1718 edges (Supplementary Data Sheet S4). The average number of neighbors, representing the average connectivity among network nodes, was 9.212, and network centralization and heterogeneity were 0.178 and 1.633, respectively, indicating the polypharmacology characteristic of TCM. These results indicated that nine herbs in QFLT acted synergistically to treat ALI/ARDS in a “multi-compound, multi-target” mode. Acacetin (degree 73, Neighborhood Connectivity 13), Baicalein (degree 71, Neighborhood Connectivity 12), Luteolin (degree 61, Neighborhood Connectivity 14), Liquiritigenin (degree 75, Neighborhood Connectivity 10), Wogonin (degree 71, Neighborhood Connectivity 13), and Isorhamnetin (degree 68, Neighborhood Connectivity 14) may be the important anti-inflammatory and anti-oxidative compounds of QFLT against ALI/ARDS. These indicated that QFLT’s anti-inflammatory and anti-oxidative pharmacological effects has correlation with regulation of disease targets based on multiple anti-inflammatory and anti-oxidative components.

### The Analyses of Critical Targets Between QFLT and ALI/ARDS

**FIGURE 5:**
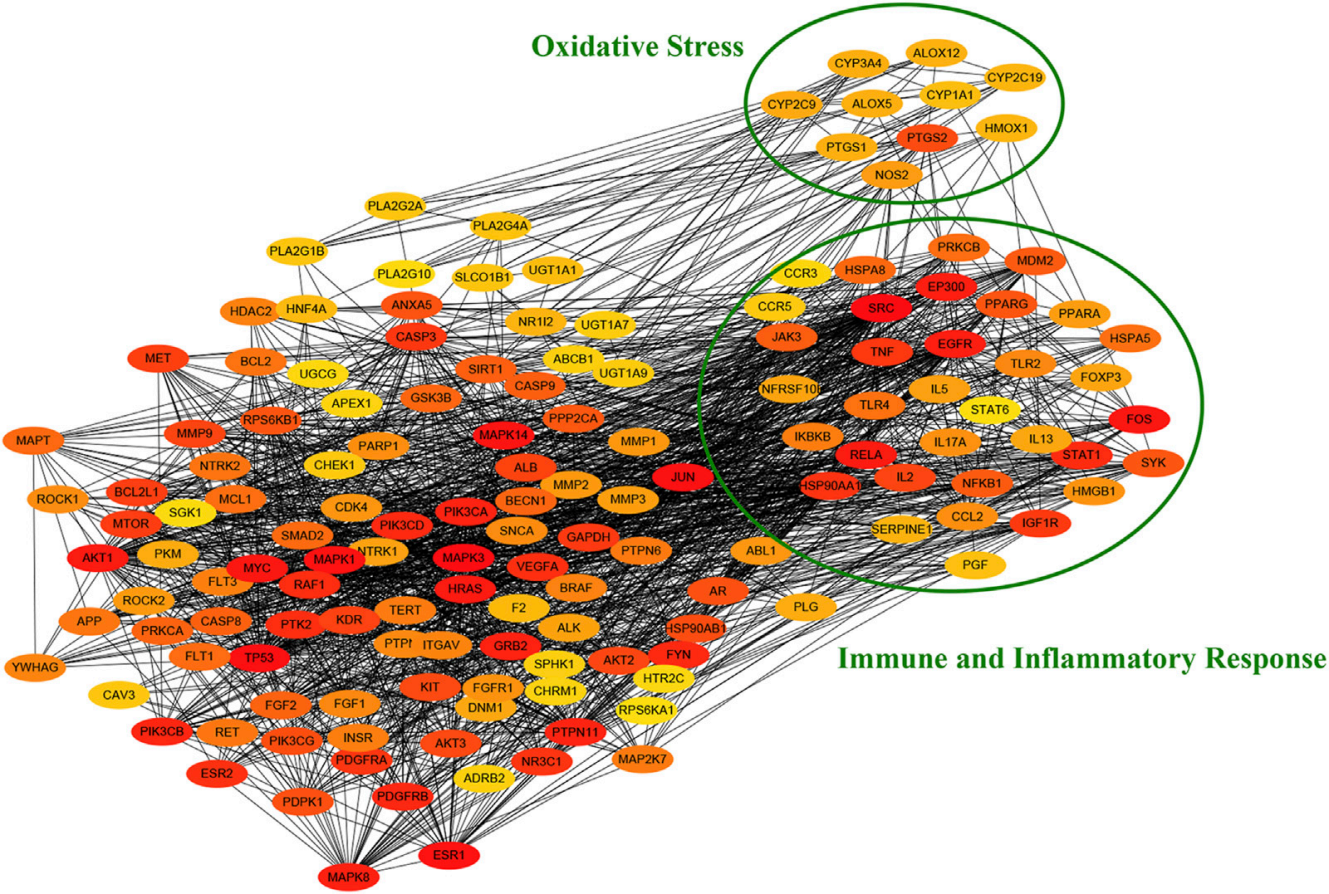
PPI network of potential targets.

To further explore the function of target protein interaction, the PPI network obtained from the STRING database was analyzed by cytohubba app of cytoscape. As shown in Figure 5, we identified a number of immune and inflammatory response (cytokine and pattern recognition receptors) and oxidative stress (oxidoreductases such as CYP450 and ALOX) related targets in the PPI network through gene annotation. The results suggested the potential pharmacological effects of QFLT in anti-infection, anti-inflammation, anti-oxidant, alleviation of cytokine storm and organism impairment.

### The Analyses of Enrichment of GO and KEGG Pathways

**FIGURE 6:**
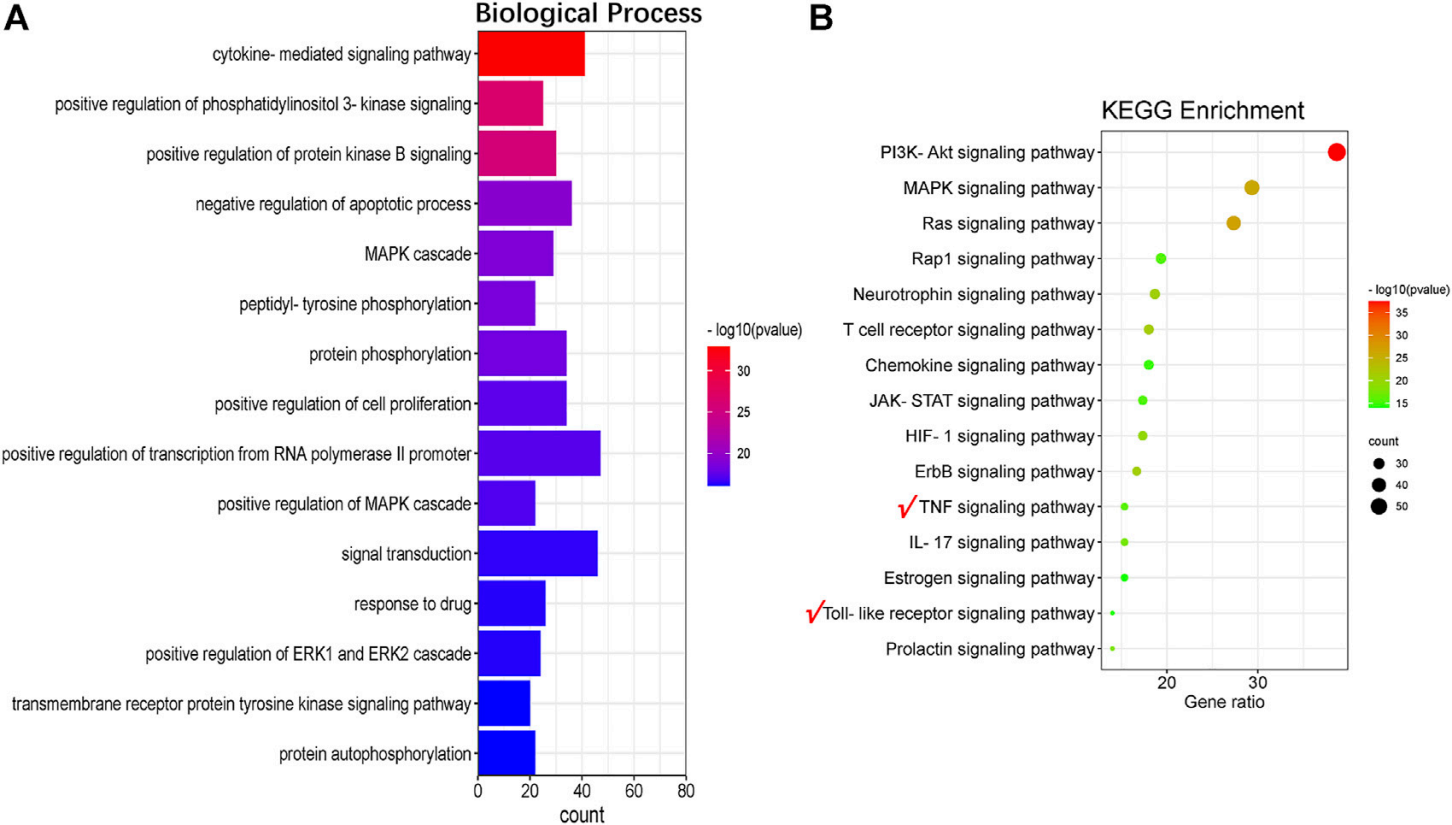
Go analyses **(A)** and KEGG enrichment **(B)**.

To further explore the anti-inflammatory and anti-oxidative functions of QFLT in ALI/ARDS, we performed a potential GO and KEGG pathways enrichment analysis on the critical targets via DAVID. Figure 6A showed results of the top 15 GO analyses (adjusted *p* < 0.01), suggesting that many biological processes may be involved in ALI/ARDS treatment, including cytokine-mediated signaling pathway, positive regulation of phosphatidylinositol 3-kinase signaling, and positive regulation of protein kinase B signaling.

For concise presentation, the top 15 of KEGG pathways (adjusted *p* < 0.01) were presented on bubble charts after removing human disease pathways. As shown in Figure 6B, these pathways may be the crucial pathways for ALI/ARDS treatment. The important targets of immune and inflammatory response such as TNF, RELA, NFKB1, IKBKB, and TLR4 were enriched in the TNF signaling pathway and Toll-like receptor signaling pathway. The activation of TNF signaling pathway plays an integral role in the production of proinflammatory cytokines such as IL-1β, TNF-α, and IL-6 (Walczak, 2011). TLR4 is a susceptibility gene for ALI, and TLR4-TRIF-TRAF6 signaling was a critical pathway of ALI. The production of oxidants can trigger lung injury and stimulate cytokine production by lung macrophages via TLR4-TRIF, suggesting that oxidative stress and innate immunity playing critical roles in ALI (Imai et al., 2008). Therefore, we will further focus on TNF signaling pathway, Toll-like receptor signaling pathway and verify the downstream biomarkers (TNF-α, IL-1β, IL-6) *in vivo* and *in vitro* experiments.

### QFLT Alleviated Pulmonary Morphological Damage in LPS-Challenged ALI Mice

**FIGURE 7:**
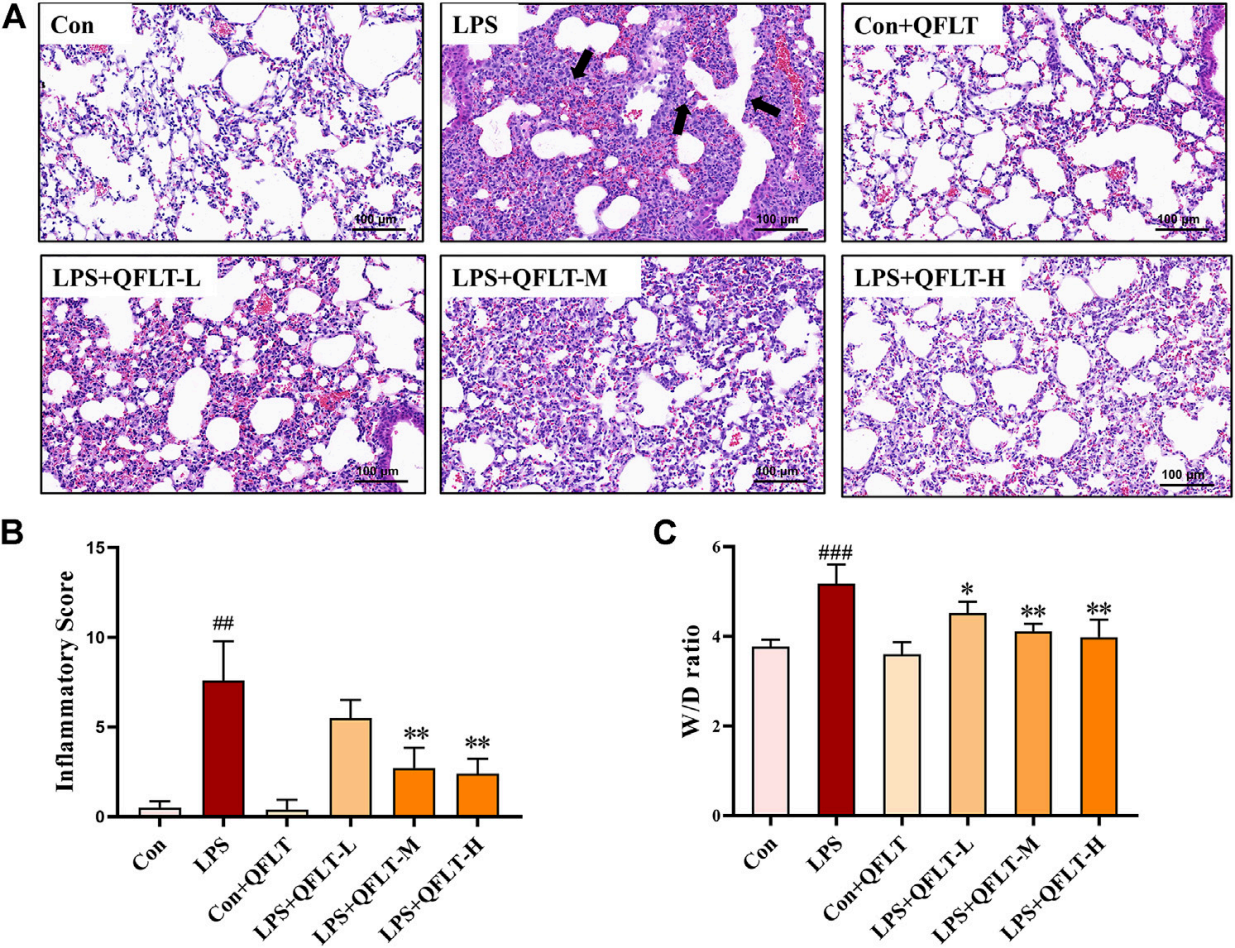
The effect of QFLT treatment against LPS-induced acute lung injury. QFLT (4, 8, and 16 g/kg) was administered to mice once daily for 7 days before intratracheal instillation of LPS (5 mg/kg). 24 h later, all mice were anesthetized and sacrificed. **(A)** Representative histological H&E-stained lung sections (×100). The black arrow indicated the alveolar structure was severely damaged, and the alveolar cavity hemorrhages with inflammatory cell infiltration. **(B)** Inflammatory Score **(C)** W/D ratio were determined at 24 h after LPS challenge. The values represented the mean ± SD (*n* = 5). ^##^ *p* < 0.01 vs. the control group; ^*^ *p* < 0.05 vs. the LPS group.

The lung W/D ratio was significantly increased in the LPS group compared to the control group (*p* < 0.001). However, QFLT reversed this trend dose-dependently compared with the LPS group (*p* < 0.05). As shown in Figure 7B, QFLT decreased the inflammatory score of ALI lung tissue compared with the LPS group. For the H&E staining, as show in Figures 7A–C, the alveolar structure of the LPS group was severely damaged, alveolar cavity hemorrhage accompanied by inflammatory cell infiltration, pulmonary interstitial edema, and the alveolar septum was significantly thicker than the control group. QFLT significantly alleviated the pulmonary morphological damage challenged by LPS.

### QFLT Treatment Reduced Total Cell Number, Macrophages and Neutrophils in LPS-Challenged ALI Mice

**FIGURE 8:**
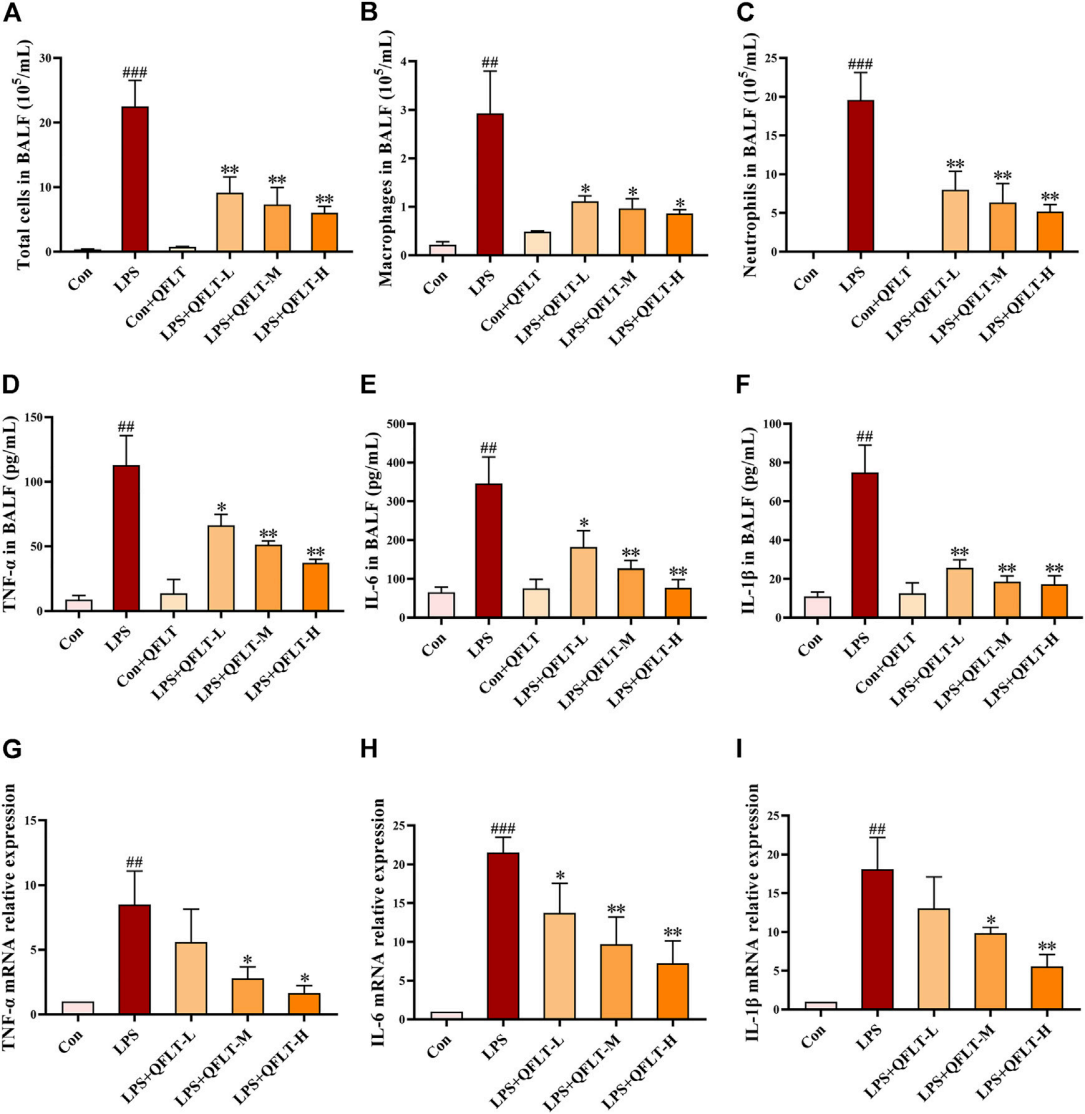
Effects of QFLT treatment on inflammatory levels *in vivo*. **(A–C)** After staining with Wright–Giemsa solution, the number of total cells, neutrophils, and macrophages in BALF were counted using light microscopy. **(D–F)** The levels of TNF-α, IL-1β and IL-6 in BALF were significantly inhibited by QFLT. **(G–I)** Levels of TNF-α, IL-1β and IL -6 in LPS-challenged mice lung tissue measured by qPCR. Effects of QFLT treatment on levels of oxidation product *in vivo*. Data were expressed as the mean ± SD (*n* = 3). ^##^ *p* < 0.01 vs. the control group; ^*^ *p* < 0.05 vs. the LPS group.

Total cells, neutrophils and macrophages contained in BALF from mice exposed to LPS were counted and stained with Wright-Giemsa. As shown in Figures 8A–C, compared with the control group, the numbers of total cells, neutrophils and macrophages were significantly increased in the LPS group (*p* < 0.01), and QFLT pretreatment significantly decreased the number of inflammatory cells compared with LPS group (*p* < 0.05).

### Treatment With QFLT Suppressed Levels of Proinflammatory Cytokines *In Vivo*

To determine whether QFLT regulated inflammatory and immune response in LPS-challenged mice, we collected BALF to examine proinflammatory cytokines in the alveolar microenvironment. As illustrated in Figures 8D–F, compared with the control group, secretions of TNF-α, IL-6, and IL-1β were elevated significantly in LPS group (*p* < 0.01), which was significantly inhibited by QFLT treatment (*p* < 0.05). In Figures 8G–I, levels of *TNF-α*, *IL-6,* and *IL-1β* mRNA significantly increased in LPS-challenged mice lung tissue (*p* < 0.01), and QFLT treatment significantly suppressed the mRNA level of these genes (*p* < 0.05).

### QFLT Decreased the Levels of Proinflammatory Cytokines Secreted by LPS-Activated Macrophages

**FIGURE 9:**
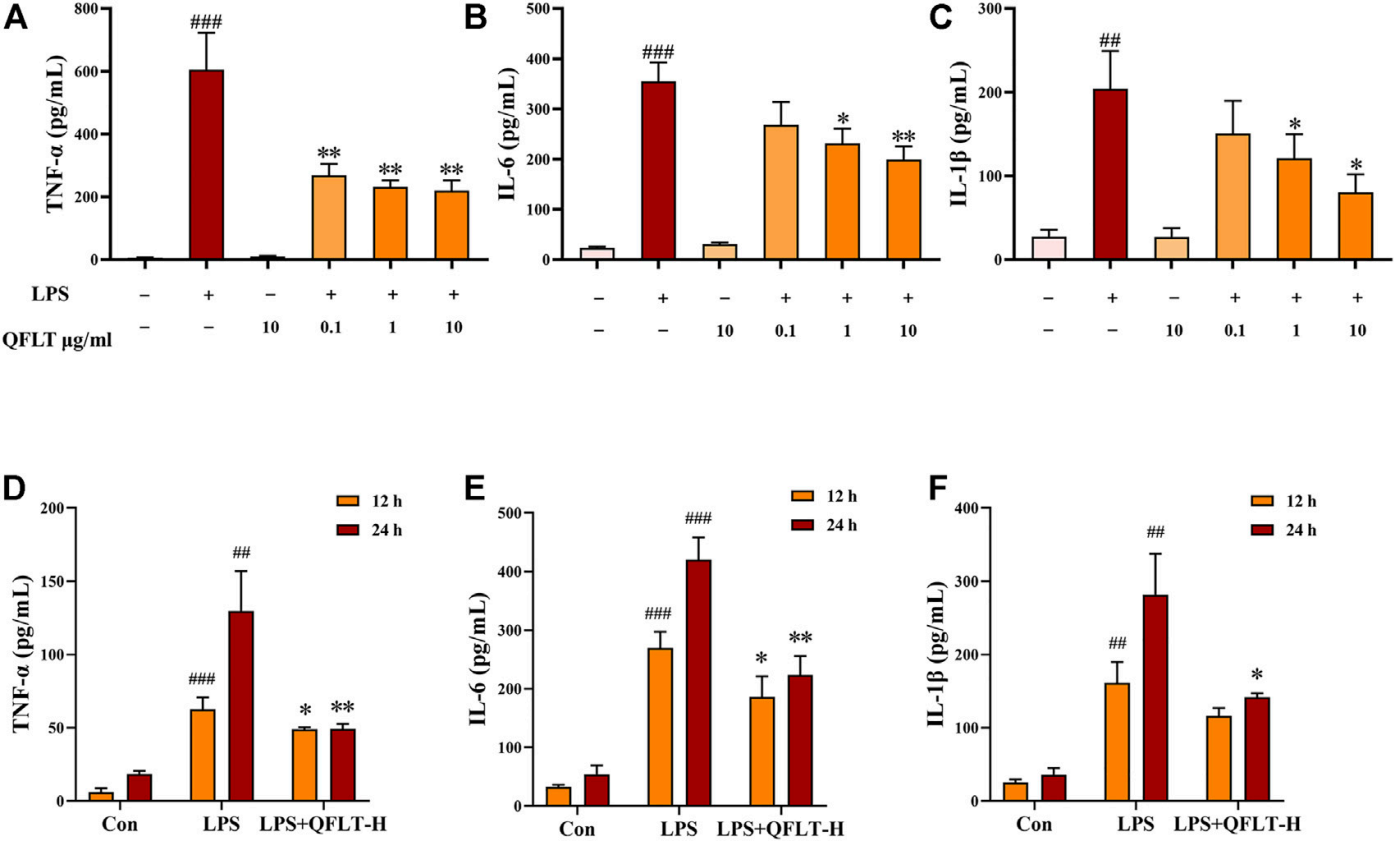
Effects of QFLT treatment on secretion of proinflammatory cytokines (TNF-α, IL-1β and IL-6) of LPS-activated macrophages *in vitro* **(A–F)**. Macrophages were incubated with/without LPS (5 μg/ml) in the absence or presence of QFLT (0, 0.1, 1, 10 μg/ml) for 24 h. Then the supernatant was detected using an ELISA kit. Data were expressed as the mean ± SD (*n* = 3). ^##^ *p* < 0.01 vs. the control group; ^*^ *p* < 0.05 vs. the LPS group.

Macrophages, as the primary contributors, taking essential responsibilities to potentially pathological inflammatory processes, can produce rapidly large amounts of inflammatory cytokines in response to danger signals (Hamidzadeh et al., 2017). To confirm the anti-inflammatory pharmacological effects of QFLT in LPS- activated macrophages, macrophages were incubated with or without LPS (5 μg/ml) in the absence or presence of different concentrations of QFLT (0, 0.1, 1, 10 μg/ml) for 24 h. The levels of TNF-α, IL-6, and IL-1β were detected by ELISA (Figures 9A–C). The results showed that compared with the LPS group, the levels of TNF-α, IL-6, and IL-1β were also significantly reduced by QFLT treatment in a dose-dependent manner (*p* < 0.05). In Figures 9D–F, we also further explored whether QFLT treatment was time dependent in changes of proinflammatory cytokines levels. The levels of TNF-α, IL-6, and IL-1β were measured in LPS-activated macrophages after 12-h and 24-h of QFLT treatment, respectively. The results showed that QFLT can decrease the levels of TNF-α, IL-6 and IL-1β in LPS-activated macrophages in a time-dependent manner (*p* < 0.05).

### QFLT Treatment Alleviated Levels of Oxidative Stress *In Vivo*

**FIGURE 10:**
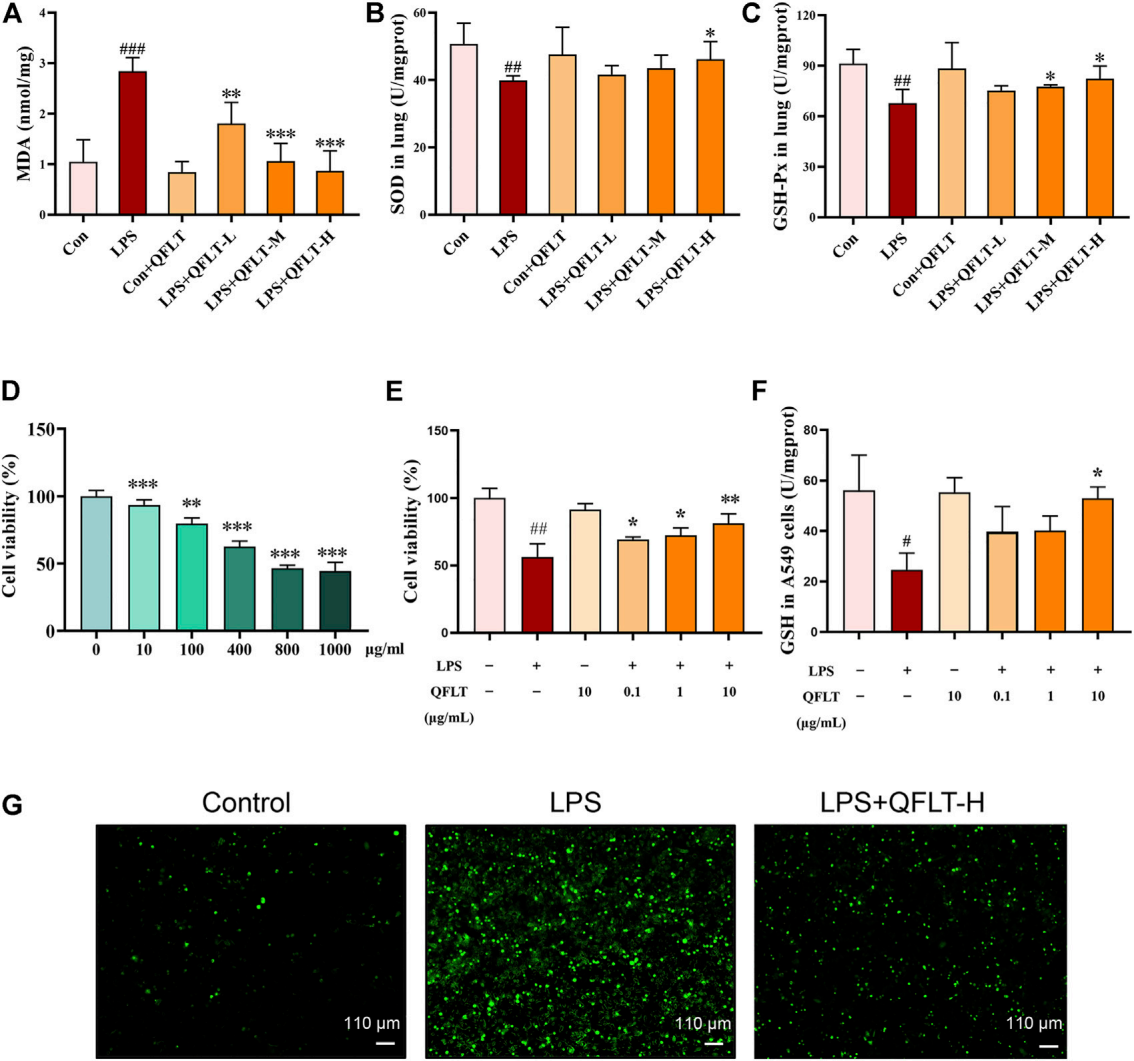
Effects of QFLT treatment on oxidative stress *in vivo* and *in vitro*. QFLT treatment reduced the lung tissue levels of MDA, and levels of GSH-Px and SOD depletion in LPS-challenged mice **(A–C)**. Effects of QFLT treatment on LPS-activated cell viability and oxidation production *in vitro* **(D–G)**. **(D)** Different concentrations (0–1,000 μg/ml) of LPS-activated A549 cells. **(E)** A549 cells were in the absence or presence of different concentrations with QFLT (0, 0.1, 1, 10 μg/ml) pretreatment for 24 h and then incubated with/without LPS (800 μg/ml) for 24 h **(F)** QFLT pretreatment may increase GSH levels in LPS-activated A549 cells **(G)** QFLT pretreatment reduced ROS generation in LPS-activated A549 cells as detected using a ROS kit (×4). Data were shown as the mean ± SD (*n* = 3). ^#^ *p* < 0.05 vs. the control group; ^*^ *p* < 0.05 vs. the LPS group.

To further investigate the potential role of QFLT in ALI/ARDS as an antioxidant, we further examined multiple oxidative stress-related biomarkers. MDA can reflect the severity of oxidative stress damage, while SOD and GSH-Px can reduce tissue and cell damage caused by oxidative stress. Compared with the control group, the LPS group of mice showed increased MDA level (*p* < 0.001) but decreased SOD (*p* < 0.01) and GSH-Px level (*p* < 0.01). The results demonstrated that QFLT treatment significantly attenuated MDA generation and SOD and GSH-Px depletion (*p* < 0.05) (Figures 10A–C).

### QFLT Affected LPS-Activated A549 Cells Viability and Oxidation Products in Lung Epithelial A549 Cells

Oxidant production within lung can lead to extensive destruction of alveolar epithelial cells in ALI/ARDS (Ward, 2010). Therefore, we sought to determine the effects of QFLT treatment on LPS- activated alveolar epithelial A549 cells. To determine the proper LPS concentration on the oxidation model of alveolar epithelial A549 cells, the cells were incubated with different concentrations of LPS (0–1,000 μg/ml) for 24 h, then the cell viability was assessed by CCK-8 assay. Accordingly, A549 cells were in the absence or presence of different concentrations with QFLT (0, 0.1, 1, 10 μg/ml) pretreatment for 24 h and then incubated with/without LPS (800 μg/ml) for 24 h. The results suggested that QFLT pretreatment enhanced LPS-activated A549 cell viability in a concentration-dependent manner (*p* < 0.05). Reduced glutathione (GSH), as an enzyme-catalyzed antioxidant, plays an important role in alleviating oxidative tissue injury. Compared with the LPS group, QFLT treatment increased GSH content (*p* < 0.05). ROS generation was assessed in LPS-activated A549 cells indicating that QFLT pretreatment dramatically reduced ROS generation (Figures 10D–G).

## Discussion

Acute lung injury and acute respiratory distress syndrome are life-threatening illnesses that are closely associated with morbidity and mortality in intensive care unit (ICU) admissions, and there is an urgent need to explore new treatments. This study combined systemic pharmacology strategies with *in vivo* and *in vitro* experiments to investigate the potential anti-inflammatory and anti-oxidative mechanisms of QFLT as a treatment for ALI/ARDS. Furthermore, systemic pharmacology analysis reflected the holism of TCM treatment, providing a new strategy for research on TCM prescriptions.

The potential mechanism of the anti-inflammatory and anti-oxidative effects of QFLT was explored by using network pharmacological analysis based on UHPLC-MS. UHPLC-MS identified 47 active components including 35 flavonoids, which exhibited anti-oxidative, radical scavenging, and anti-inflammatory biological activity *in vitro* and *in vivo* (Panche et al., 2016; Singh et al., 2020). Based on the network analysis, the essential anti-inflammatory and anti-oxidative components of QFLT may be Acacetin, Baicalein, Luteolin, Liquiritigenin, Wogonin, and Isorhamnetin. These natural compounds have been found to have potential benefits in ALI therapy (He et al., 2021). Acacetin showed protective effects in sepsis-induced ALI, decreased iNOS and COX-2 expression, and increased HO-1 expression and SODs activity (Sun et al., 2018; Wu et al., 2018). Baicalein dampened the NF-κB, MAPK and STAT3 signaling pathways to exert its anti-inflammatory effects (Pu et al., 2019), and abrogated ROS-mediated mitochondrial dysfunction *in vivo* (Naveenkumar et al., 2013). Liquiritigenin attenuated inflammatory effects by inhibition of NFκB activation in macrophages, decreasing production of iNOS and proinflammatory cytokines (Kim et al., 2008). Luteolin may be effective for treatment of lung injury by preventing NF-κB activation and activating AKT/Nrf2 pathway (Liu et al., 2018). Moreover, Luteolin elevated cellular GSH levels but reduced ROS generation (Tan et al., 2014). Wogonin suppressed IL-10 production in B cells via inhibition of the STAT3 and ERK signaling pathway (Fan et al., 2020). Isorhamnetin can inhibit the release of inflammatory mediators (TNF-α, IL-1β, IL-6, iNOS and COX-2) and MDA levels as well as enhanced SOD levels in LPS-challenged mice (Yang et al., 2016), blocking the MAPK and NF-κB signaling pathways (Li et al., 2016). These findings suggested that the flavonoids of QFLT attenuated lung damage by regulating immune process and oxidative stress.

**FIGURE 11:**
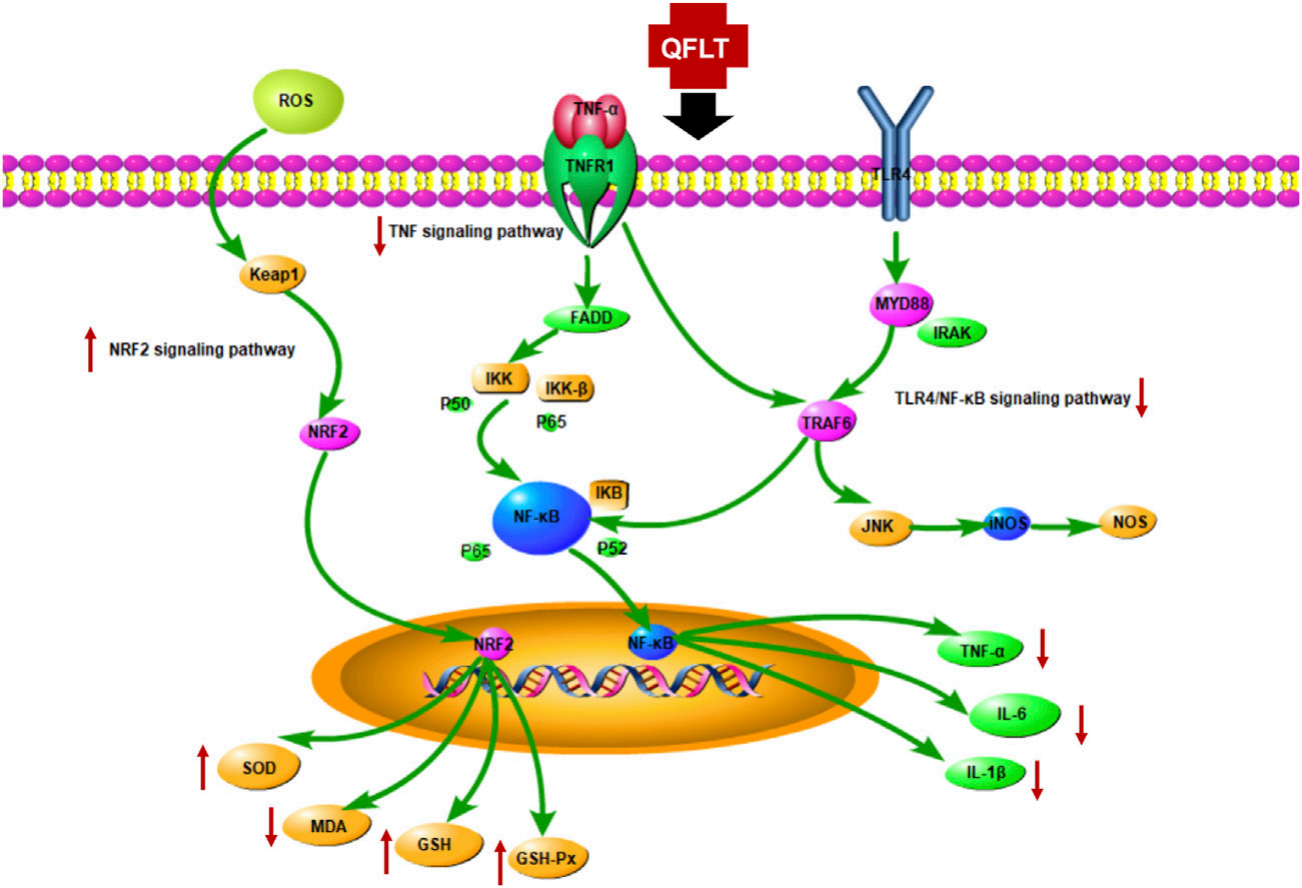
The potential anti-inflammatory and anti-oxidative mechanism of QFLT against ALI/ARDS.

KEGG enrichment analysis showed that the key pathways involved in ALI/ARDS may be mainly TNF signaling pathway and Toll-like receptor signaling pathway. Moreover, excessive inflammatory responses and disordered oxidative stress were crucial in the pathogenesis of ALI (Sarma and Ward, 2011; Matthay et al., 2012). The TNF receptor superfamily, and the Toll-Like receptor family (TLRs) mainly activated the downstream NF-κB pathway (Morgan and Liu, 2011), which has been considered the central mediator of the inflammatory process and innate and adaptive immune responses (DiDonato et al., 2012), subsequently releasing a variety of proinflammatory cytokines, such as IL1β, IL18, IL6, and TNF-α. Keap1-Nrf2 signaling was one of the most pivotal endogenous anti-oxidative stress pathway, which also is the significant target for inflammation-related disorders (Li et al., 2021). Toxic oxygen products can be generated when macrophages or PMNs undergo stimulation by factors such as bacterial LPS or IgG immune complexes (Ward, 2010). We also explored the potential role of QFLT as an antioxidant in ALI/ARDS, and examined multiple biomarkers regulating oxidative stress. Figure 11 summarizes the potential anti-inflammatory and anti-oxidative mechanisms of QFLT against ALI/ARDS: downstream biomarkers (TNF-α, IL-1β, IL-6, SOD, MDA, GSH, ROS, GSH-Px) of these pathways were verified *in vivo* and *in vitro* experiments.

In this study, QFLT treatment alleviated the pathological manifestations in ALI mice. The pathogenesis of ALI/ARDS mainly involves three phases, exudative phase, proliferative phase, and fibrotic phase (Sweeney and McAuley, 2016). During the exudative phase of ARDS, the classic characteristics of pathogenesis include innate immune cell–mediated damage of the alveolar endothelial and epithelial barriers, and accumulation of edema fluid in the interstitium and alveolus (Thompson et al., 2017). H&E staining and W/D ratio showed pulmonary interstitial edema and obvious inflammatory infiltration in the LPS group. Furthermore, the pathological changes were reversed by QFLT in a dose-dependent manner.

In the ALI/ARDS process, resident alveolar macrophages dissociate from the alveolar epithelial cells and secrete proinflammatory cytokines, leading to the recruitment of neutrophil and monocyte or macrophage (Aggarwal et al., 2014; Butt et al., 2016; Thompson et al., 2017). Subsequently, more proinflammatory cytokines and oxidation products (TNF-α, IL-1β, IL-6, and ROS) are released and cause tissue damage. Endothelial and epithelial dysfunction further result in leakage of fluids from circulation into the interstitial space and alveoli (Herold et al., 2011; Kellner et al., 2017). Furthermore, the regulation of inflammatory response and oxidative stress is particularly important in this pathological process. The total cell number, macrophages, neutrophils, and proinflammatory cytokines in BALF from LPS-challenged mice were significantly decreased, and the increase of TNF-α, IL-6, and IL-1β mRNA in lung tissue was suppressed remarkably by QFLT treatment. *In vitro*, the levels of proinflammatory cytokines TNF-α, IL-6, and IL-1β secreted by LPS-activated macrophages was decreased by QFLT treatment. In addition, QFLT treatment also decreased the content of MDA, an indicator of lung oxidative damage. SOD enzymes and GSH-Px as antioxidant systems can regulate ROS-dependent signaling and oxidant balance (Sareila et al., 2011; Lei et al., 2015). The levels of SOD enzymes and GSH-Px were increased by QFLT treatment in LPS-challenged mice lung tissues. QFLT also regulated the cells viability and oxidation products (ROS and GSH) in lung epithelial A549 cells activated by LPS. These *in vivo* and *in vitro* results showed that QFLT has anti-inflammatory and anti-oxidative pharmacological effects in the treatment of ALI/ARDS.

In summary, we explored the effects of QFLT as a treatment for ALI/ARDS treatment. The potential mechanisms of anti-inflammation and antioxidant effects were investigated through a systems pharmacology strategy. Moreover, the superior anti-inflammatory and anti-oxidative pharmacological effects of QFLT has been verified in the treatment of ALI/ARDS *in vivo* and *in vitro*. Thus, this study showed the potential of QFLT as a Chinese Medicine therapeutic strategy for ALI/ARDS. In the future studies, it is necessary for us to further verify the anti-inflammatory and anti-oxidative mechanism of QFLT in the treatment of ALI/ARDS and explore in other models.

## Data Availability

The original contributions presented in the study are included in the article/Supplementary Material, further inquiries can be directed to the corresponding authors.
